# Nine-, but Not Four-Days Heat Acclimation Improves Self-Paced Endurance Performance in Females

**DOI:** 10.3389/fphys.2019.00539

**Published:** 2019-05-16

**Authors:** Nathalie V. Kirby, Samuel J. E. Lucas, Rebekah A. I. Lucas

**Affiliations:** School of Sport, Exercise and Rehabilitation Sciences, University of Birmingham, Birmingham, United Kingdom

**Keywords:** heat acclimation, acclimatization, thermoregulation, female, women, exercise physiology, sports performance

## Abstract

Although emerging as a cost and time efficient way to prepare for competition in the heat, recent evidence indicates that “short-term” heat acclimation (<7 days) may not be sufficient for females to adapt to repeated heat stress. Furthermore, self-paced performance following either short-term, or longer (>7 days) heat acclimation has not been examined in a female cohort. Therefore, the aim of this study was to investigate self-paced endurance performance in hot conditions following 4- and 9-days of a high-intensity isothermic heat acclimation protocol in a female cohort. Eight female endurance athletes (mean ± SD, age 27 ± 5 years, mass 61 ± 5 kg, VO_2peak_ 47 ± 6 ml⋅kg⋅min^−1^) performed 15-min self-paced cycling time trials in hot conditions (35°C, 30%RH) before (HTT1), and after 4-days (HTT2), and 9-days (HTT3) isothermic heat acclimation (HA, with power output manipulated to increase and maintain rectal temperature (*T*_rec_) at ∼38.5°C for 90-min cycling in 40°C, 30%RH) with permissive dehydration. There were no significant changes in distance cycled (*p* = 0.47), mean power output (*p* = 0.55) or cycling speed (*p* = 0.44) following 4-days HA (i.e., from HTT1 to HTT2). Distance cycled (+3.2%, *p* = 0.01; +1.8%, *p* = 0.04), mean power output (+8.1%, *p* = 0.01; +4.8%, *p* = 0.05) and cycling speed (+3.0%, *p* = 0.01; +1.6%, *p* = 0.05) were significantly greater in HTT3 than in HTT1 and HTT2, respectively. There was an increase in the number of active sweat glands per cm^2^ in HTT3 as compared to HTT1 (+32%; *p* = 0.02) and HTT2 (+22%; *p* < 0.01), whereas thermal sensation immediately before HTT3 decreased (“Slightly Warm,” *p* = 0.03) compared to ratings taken before HTT1 (“Warm”) in 35°C, 30%RH. Four-days HA was insufficient to improve performance in the heat in females as observed following 9-days HA.

## Introduction

Hot ambient temperatures and elevated humidity are known to negatively impact endurance exercise performance (Tatterson et al., 2000; Périard et al., 2011). Heat acclimation is an effective strategy to drive favorable physiological adaptations, thereby reducing athletic performance impairments caused by these challenging environments (Sawka et al., 2011; Périard et al., 2015; Racinais et al., 2015). Heat acclimation typically consists of repeated daily heat stress exposures, with exposure durations commonly lasting between 60 and 90 min. Traditionally, 10 days of heat exposure are undertaken to elicit the heat acclimation phenotype and improve endurance performance in the heat (Armstrong and Maresh, 1991; Lorenzo et al., 2010; Sawka et al., 2011), though 75–80% of physiological adaptations occur in the first 4–7 days of heat acclimation in male cohorts (Pandolf, 1998; Shapiro et al., 1998). Based on this, Garrett et al. (2009) first demonstrate meaningful performance improvements following just 5 days of isothermic heat acclimation, termed “short-term heat acclimation” (STHA).

STHA has since been defined as being <7 days in length (Garrett et al., 2011), and is promoted as a cost and time efficient option for athletes preparing for competition in the heat. Successful STHA lasting 4–7 days in male cohorts has been well documented (Petersen et al., 2010; Fujii et al., 2012; Chen et al., 2013; Garrett et al., 2012, 2014; Best et al., 2014; Costa et al., 2014; Gibson et al., 2015; Mee et al., 2015; Racinais et al., 2015; Guy et al., 2016; James et al., 2016; Willmott et al., 2016). However, few studies to date have examined STHA effects in female cohorts. It was initially shown that there were no sex differences (4 females vs. 4 males) in adaptations to 10-days heat acclimation when aerobic fitness and surface area to mass ratios were matched (Avellini et al., 1980). However, Mee et al. (2015) more recently reported a significant sex difference in the time course of heat acclimation (i.e., 5- vs. 10-days), challenging past assumptions. In this study Mee et al. (2015) found that following STHA females did not exhibit a lower resting core temperature, or an attenuated rise in core temperature and heart rate (HR) when exercising at a fixed workload, critical requirements in demonstrating the heat acclimation adaptation. However, the male cohort successfully attained these adaptations following the same protocol. Mee et al. (2018) then demonstrated that a longer daily heat exposure (achieved via 20-min of sitting in sauna suits in 50°C, 30%RH immediately before 90-min isothermic heat acclimation) successfully induced heat adaptations in a female cohort following STHA (5-days). The authors therefore concluded that females require either a longer daily heat exposure (Mee et al., 2018), or a greater number of heat exposures (Mee et al., 2015) to elicit favorable physiological adaptations.

It is unclear if females can achieve meaningful performance improvements following STHA or if a longer heat acclimation period is needed. Sunderland et al. (2008) reported a 33% improvement in distance run during a repeated shuttle run performance test following STHA in a female cohort as well as a reduced rate of rise in rectal temperature (*T*_rec_). The authors attributed performance improvements to the high-intensity intermittent exercise performed during the acclimation sessions, a strategy implemented by Pethick et al. (2018) to successfully induce plasma volume expansion in a female cohort following 5-days high-intensity heat acclimation. Therefore, the addition of high-intensity interval training (HIIT) may offer a means to increase effectiveness of STHA in a female cohort. However, the performance test employed by Sunderland and colleagues was a time to exhaustion trial, which is a less reliable test and subject to greater variation than self-paced performance tests (Hopkins et al., 2001; Borg et al., 2018). Self-paced performance outcomes and their improvements following heat acclimation have only been documented in male (Garrett et al., 2012; Keiser et al., 2015; Racinais et al., 2015; Guy et al., 2016; Wingfield et al., 2016) or mostly male (Lorenzo et al., 2010) cohorts, and remain to be investigated in females. Such information is timely for female athletes competing at upcoming international competitions in hot climates, such as the 2020 Olympic Games in Tokyo and the 2019 IAAF World Athletics Championships in Doha. Currently, female athletes must either depend on conflicting literature or fill knowledge gaps with information inferred from male cohorts.

Therefore, the aim of the present study was to investigate self-paced endurance performance in hot conditions following 4-days (STHA), and 9-days of a high-intensity isothermic heat acclimation protocol in a female cohort. It was hypothesized that females would not exhibit performance improvements in self-paced exercise following STHA, as previous studies indicate that 4-days of 90-min heat acclimation is unlikely to be a sufficient stimulus for the thermoregulatory and cardiovascular adaptations necessary for performance improvements in the heat. We further hypothesized that performance improvements in power output and time trial distance would occur following 9-days heat acclimation.

## Materials and Methods

### General Overview and Design

**FIGURE 1:**
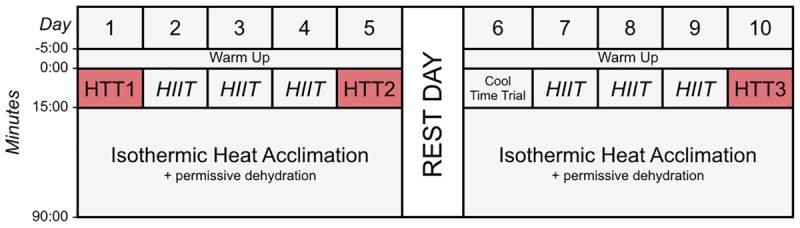
Schematic diagram of the time trials and heat acclimation sessions (HA). Time trials were conducted in hot conditions [HTT; 35°C, 30% relative humidity (RH)], before HA (HTT1), after 4-days (HTT2) and after 9-days (HTT3) HA. On days 2–4 and 7–9, participants completed 15-min of high-intensity intervals (HIIT), where maximum effort was given for 15-s, with 45-s of active recovery. Participants then undertook 75-min of isothermic heat acclimation (where exercise intensity was manipulated to increase and maintain rectal temperature at ∼38.5°C; 40°C, 30%RH) with permissive dehydration. There was one rest day following 5-days HA. Cool Time Trial refers to a 15-min cycling time trial in cool conditions (15°C, 30% RH), which was part of a larger dataset that are not reported herein.

This study was approved by the University of Birmingham Ethics Committee, and conformed to the standards set by the Declaration of Helsinki 2013. All participants were informed of the experimental procedures and possible risks involved in the study before their written consent was obtained. Each participant completed a general exercise questionnaire and a menstrual cycle questionnaire (detailing the day their menstrual cycle commenced, premenstrual symptoms, and contraceptive medication or devices) to ascertain what phase of their cycle they were in for each time trial. All experimental procedures were completed in the environmental chamber (TIS Services, Hampshire, United Kingdom) in the School of Sport, Exercise and Rehabilitation Sciences building at the University of Birmingham. Participants performed all heat acclimation and testing sessions at the same time of day (±2 h), and at similar times to their normal training sessions so as not to disrupt their normal circadian rhythms (Reilly and Brooks, 1986). This included mornings, afternoons, or evenings. Participants were familiarized with the 15-min time trial performance tests in cool conditions (15°C, 30% RH) on three occasions, with the final occasion 48 h prior to beginning the protocol. Participants performed a 15-min cycling time trial in hot (35°C, 30% RH) conditions pre-acclimation, following 4-days (STHA), and following 9-days isothermic heat acclimation (HA). An overview of the hot time trials and HA sessions are displayed in Figure 1. This experiment was conducted in the United Kingdom during the months of February, April, May, and June, when mean ambient temperatures were below 20°C (exclusive of 3 days where the mean daily temperatures were 23, 24, and 27°C, respectively). The protocol was performed in addition to normal training sessions (i.e., weight training and normal conditioning such as swimming and running). Participants’ activity was not restricted, except on the day prior to (no exhaustive exercise) or the day of (no other activity) time trials in hot conditions (HTTs). Participants were asked to refrain from alcohol and overly strenuous exercise outside of the laboratory 48 h before time trials.

### Participants

Eight recreational endurance athletes aged 21–35 years volunteered for and completed this study. An additional participant volunteered, but dropped out due to relocation after preliminary testing and was not included in the results. All participants were familiar with competitive, race-style endurance events, and trained 5 ± 1 days per week, averaging 9 ± 4 h of weekly endurance exercise training. Participants were eumenorrheic or using various forms of hormonal contraceptives (Table 1) and did not report any negative premenstrual symptoms that may have affected performance during time trials (Giacomoni et al., 2000). Participants had not previously undergone a heat acclimation protocol and had not been in hot conditions for the past 2 months. Participants also completed an incremental (20 W ⋅ min^−1^ stages) exercise test on a cycle ergometer (Sport Excalibur, Lode, Groningen, Netherlands) to determine maximal aerobic capacity (VO_2peak_), with expired air (Vyntus CPX, Jaeger, Wuerzberg, Germany) and HR (Polar Electro, Kempele, Finland) measured continuously. Personal characteristics are summarized in Table 1.

**Table 1:** Participants’ personal characteristics.

Participant	Age (years)	Height (cm)	VO_2peak_ (ml ^⋅^ kg^−1^ ⋅min^−1^)	Body mass (kg)	Menstrual cycle/contraceptive	Day of menstrual cycle or pill taking phase on HTT1
1	32	168	40	54	OCP (Cilest)	15
2	28	168	45	59	Implant	N/A
3	25	176	43	65	Implant	N/A
4	23	165	43	55	EU	23
5	35	173	42	69	EU	17
6	32	172	53	63	IUD Coil	N/A
7	23	165	53	61	OCP (Yasmin)	11
8	21	174	54	61	Implant	N/A
*Mean*	27	170	47	61		

*OCP, oral contraceptive pill user (pill brand); IUD Coil, copper coil intrauterine device; EU, eumenorrheic natural cycle.*

### Heat Acclimation Sessions

**FIGURE 2:**
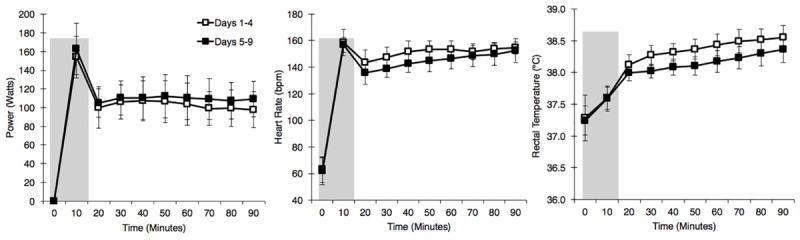
Power output **(left)**, heart rate **(middle)**, and rectal temperature **(right)**, at 10 min intervals during the heat acclimation protocol. Open squares represent mean ± SD of data from days 1 to 4, and solid squares represent mean ± SD of data from days 5 to 9. Shaded area represents a 15-min time trial or 15-min high-intensity intervals.

A combination of HIIT, permissive dehydration and isothermic heat acclimation (Garrett et al., 2014; Sunderland et al., 2008) was used to construct a “high intensity” HA protocol. Participants voided their bladder upon arrival to the laboratory to provide a urine sample. Towel-dried, nude body mass (NBM) was recorded to 0.1 kg using digital scales (Seca 877, Seca, Hamburg, Germany) before and immediately after each session to estimate sweat loss. Conditions during HA sessions were set to 40°C, 30%RH with a fan-generated airflow of ∼3 m second^−1^ facing participants. All heat acclimation sessions and time trials were completed using a cycle ergometer (Velotron, RacerMate Inc., Seattle, WA, United States), which was calibrated according to manufacturer instructions for the chosen temperature and confirmed to exhibit <1% deviation from calibration settings before each use. Following a 5-min, self-selected warm-up, participants completed 15-min of high-intensity intervals, where participants were asked to give maximum effort for 15-s, with 45-s of active recovery. The aim of the high-intensity intervals was to rapidly increase *T*_rec_. This was followed by an additional 75-min of continuous cycling at an intensity manipulated with the aim to further increase *T*_rec_ and maintain it at ∼38.5°C (Patterson et al., 2004; Garrett et al., 2012), totalling 90-min HA plus 5-min warm up. On days that hot time trials (HTT) preceded HA sessions, the HTTs were used in place of the high-intensity intervals. On these test days, the temperature of the environmental chamber was immediately increased to 40°C, 30%RH following the time trial. There was one rest day following 5-days HA. Cool Time Trial refers to a 15-min cycling time trial in cool conditions (15°C, 30% RH), which was part of a larger dataset that are not reported herein. Power output, HR, and *T*_rec_ across HA sessions during STHA (days 1–4) and days 5–9 are depicted in Figure 2. Ratings of perceived exertion (RPE; Borg, 1982), thermal sensation, and thermal comfort were recorded at 15-min intervals during HA sessions. Participants were instructed to refrain from fluid consumption as much as could be tolerated during HA sessions to induce the added stressor of dehydration (permissive dehydration; Garrett et al., 2014). Fluid consumed (295 ± 235 ml each session) was recorded by weighing water bottles to 0.001 L (Oertling, United Kingdom) before and after HA sessions, and was considered in the calculations of total body sweat loss. Heat acclimation involved 9 consecutive days of HA sessions, except for 1 day of rest following STHA (Figure 1).

### Hot Time Trials

Time trials were performed in hot conditions (35°C, 30%RH with a fan-generated airflow of ∼3 m ⋅ second^−1^ facing participants) on the 1st day of HA (Day 1; HTT1), and following 4-days HA (Day 5; HTT2), and 9-days HA (Day 10; HTT3). Participants were instructed to maintain normal hydration before each HTT, which was verified with a urine osmolality value of ≤700 mOsm ⋅ kg^−1^ (Sawka et al., 2007). Participants lay supine for 10 min of stabilization at room temperature prior to each trial to collect resting measures of *T*_rec_ and blood lactate.

Participants entered the environmental chamber and commenced a 5-min warm up at a self-selected pace, before completing a 15-min, self-paced cycling time trial. Power output and distance cycled were recorded continuously by the Velotron Coaching Software. Participants were aware of the time elapsed, as displayed by a stop-clock mounted to the handles of the cycle ergometer, however, they were blinded to any other physiological or performance feedback (i.e., HR, power output, distance cycled, etc.). Participants were given equal verbal encouragement by the same researchers at similar time points during the HTT. Free drinking was permitted during HTTs. RPE, blood lactate and sweat gland activity were recorded immediately following the HTTs. Ratings of thermal comfort and thermal sensation were reported inside the environmental chamber, preceding the warm-up for HTTs, as well as immediately after. Following HTTs, participants completed 5 min of self-paced active recovery before proceeding with the acclimation session for that day.

### Measures

Urine osmolality was measured prior to each experimental session to assess hydration (Osmocheck, Vitech Scientific Ltd., West Sussex, United Kingdom). *T*_rec_ was measured using a rectal thermistor inserted 10 cm past the anal sphincter prior to beginning each experimental session (Mon-a-Therm, Covidien, Mansfield, MA, United States). Weighted mean skin temperature (*T*_sk_) was recorded using skin thermistors (Squirrel Thermal Couples, Grant Instruments, Cambridge, United Kingdom) attached to four sites: the mid-point of the right pectoralis major (*T*_chest_), midpoint of the right biceps brachii (*T*_arm_), right rectus femoris (*T*_thigh_), and right gastrocnemius lateral head (*T*_lowerleg_). Skin and rectal thermistors were connected to a Squirrel Data Logger (Squirrel 2020 series, Eltek, Ltd., United Kingdom) and were recorded at 30-s intervals throughout HA sessions and HTTs. HR (Polar Electro, Kempele, Finland) was also recorded throughout each session. Power output and distance cycled were recorded by the Velotron Coaching Software (Velotron CS 2008, RacerMate Inc., Seattle, WA, United States). Blood lactate measures were taken from a finger-tip blood sample and immediately analyzed using a Lactate Plus analyzer (Lactate Plus, Nova Biomedical, Waltham, MA, United States). Active sweat glands were quantified using a modified-iodine paper technique with computer aided analysis (Gagnon et al., 2012). Samples were collected from the dorsal side of the thickest segment of the forearm. Thermal sensation and thermal comfort ratings were measured using 13-point and 10-point scales, respectively, which were modified from scales used by Gagge et al. (1967).

### Data Analysis

Mean *T*_rec_ for the final 75 min of the session, which followed the 15-min high-intensity intervals, is represented by *T*_rec75_. Maximum *T*_rec_ recorded during the session (Max *T*_rec_) was used to calculate *T*_rec_ increase from rest (Δ*T*_rec_). *T*_sk_ was calculated as a weighted average according to Ramanathan (1964):

$$T_{\text{sk}}=0.3\cdot (T_{\text{chest}}+T_{\text{arm}})+0.2\cdot (T_{\text{thigh}}+T_{\mathrm{lower leg}})$$

Estimated sweat rate relative to body surface area (SR_BSA_) was calculated from changes in NBM pre- to post-session with considerations of water consumed body surface area [(BSA); calculated using the formula derived by Du Bois and Du Bois, 1916] and normalized for exercise time:

$$\begin{array}{c} \mathrm{Estimated sweat loss}\;(g)=(\mathrm{pre-trial NBM}-\mathrm{post-trial}\\ \;\mathrm{NBM})+(\mathrm{water bottle pre-trial}-\mathrm{water}\\ \;\mathrm{bottle post-trial}) \end{array}$$

$$\text{BSA}({\text{m}}^{2})=0.007184\cdot ({\text{height}}^{0.725}\cdot {\mathrm{body mass}}^{0.425})$$

$$\begin{array}{c} {\mathrm{SR}}_{\mathrm{BSA}}\;(g\cdot h^{-1}\cdot m^{-2})\\ \;=(\mathrm{estimated sweat loss})\cdot {\left(1\;h\cdot {\mathrm{exercise time}}^{-1}\right)}^{-1}.\\ \;{\left(\mathrm{BSA}\right)}^{-1} \end{array}$$

Two values were obtained for measurements of resting blood lactate and an additional two values were obtained for blood lactate immediately following HTTs. The results were averaged to yield a single value for each time point (pre- and post-trial). Extreme outliers falling outside the physiological range were excluded, and only the rational value was used (Goodwin et al., 2007; *n* = 3 incidences).

Power output (watts) was recorded each second during HTTs, and an average of each minute’s power output was used to calculate area under the curve (AUC; Pruessner et al., 2003). AUC was also calculated for *T*_rec_ (recorded at 30-s intervals) during HTTs. All data were analyzed using SPSS statistical software (SPSS version 24.0.0, SPSS, Chicago, IL, United States). To assess performance and physiological differences during HA days 1–4 vs. days 5–9, a mean value was calculated for each participant across the aforementioned days, and analyzed using a repeated-measures one-way analysis of variance (ANOVA). Mean performance values during HTTs (i.e., power output and speed), AUC comparisons (power output and *T*_rec_), distance cycled, and physiological measures between HTT1, HTT2, and HTT3, were also analyzed using a repeated-measures one-way ANOVA. Additionally, 1 min averages of power output were analyzed using a two-way repeated-measures ANOVA (3 HTT × 15 time points). Normality of the data was assessed using Mauchly’s test of sphericity, and Greenhouse–Geisser corrections were applied where assumptions of sphericity were violated. When a significant main effect was found, Bonferroni-corrected *post hoc* comparisons were made. Main effect sizes for both one-way and two-way ANOVAs were calculated using partial eta-squared ($\eta_{p}^{2}$), with $\eta_{p}^{2}$ > 0.06 representing a moderate difference and $\eta_{p}^{2}$ > 0.14 representing a large difference (Cohen, 1988). To assess ordinal data (i.e., RPE, thermal sensation and thermal comfort) differences during HA days 1–4 vs. days 5–9, and between HTT1, HTT2, and HTT3, Friedman’s test was performed with *post hoc* analysis by Wilcoxon sign-rank tests. Absolute data are expressed as mean ± standard deviation (SD) and mean within-subject differences are presented with 95% confidence limits (mean difference, 95% CL: lower limit, upper limit). Significance was set at *p* < 0.05 for each analysis. A power analysis indicated that eight participants were a sufficient sample size to detect an 8–10% difference in power output during time trial performance (as observed by Lorenzo et al., 2010; Keiser et al., 2015). This analysis used an accepted parameter of power (β ≥ 0.80) at an α level of 0.05.

## Results

### Heat Acclimation Sessions

Mean *T*_rec75_ (−0.2°C, [−0.1, −0.3]; *p* < 0.01) peak *T*_rec_ (−0.1°C, [−0.1, −0.2]; *p* = 0.01), and peak *T*_sk_ (−0.4°C, [−0.1, −0.7]; *p* = 0.01) were lower during HA sessions on days 5–9 as compared to HA sessions on days 1–4. Mean HR and percentage of age-estimated maximum heart rate (%HR_max_) (−5 beats ⋅ minute^−1^, [−1, −10]; *p* = 0.03, and −3% [−1, −5]; *p* = 0.02, respectively) were also lower during HA sessions on days 5–9 as compared to HA sessions on days 1–4. These physiological changes were present in spite of a significantly higher workload (i.e., power output) on days 5–9 as compared to HA sessions on days 1–4 (−9 W, [−3, −14], *p* = 0.01). Participants’ mean RPE, thermal sensation, and thermal comfort ratings across all HA sessions were not different (*p* > 0.05) and equalled 15 ± 2 (“Hard”), 10 ± 1 (“Hot”), and 5 ± 2 (“Uncomfortable”), respectively. There were no changes in Δ*T*_rec_ or sweat loss (*p* > 0.05). Results of HA sessions are summarized in Table 2.

**Table 2:** Performance, physiological, and psycho-physical responses during heat acclimation sessions averaged across Days 1–4 and Days 5–9.

	Days 1–4	Days 5–9
Mean power output (W)	108 ± 17	117 ± 21^∗^
Mean *T*_rec75_ (°C)	38.4 ± 0.2	38.2 ± 0.2^∗^
Resting *T*_rec_ (°C)	37.3 ± 0.4	37.2 ± 0.2
Peak *T*_rec_ (°C)	38.6 ± 0.2	38.5 ± 0.2^∗^
Δ*T*_rec_ (°C)	1.4 ± 0.4	1.2 ± 0.3
Peak *T*_sk_ (°C)	35.5 ± 0.9	35.0 ± 1.0^∗^
Mean HR (beats ⋅ minute^−1^)	150 ± 6	145 ± 6^∗^
Mean %HR_max_ (%)	78 ± 3	75 ± 4^∗^
SR_BSA_ (g ⋅ h^−1^ ⋅ m^−2^)	694 ± 105	705 ± 143
Sweat loss (%BM)	2.9 ± 0.5	2.9 ± 0.6

*Data are presented as mean ± SD. T_rec_, rectal temperature; T_rec75_, mean rectal temperatures recorded during the final 75-min of the session; ΔT_rec_, maximal change in rectal temperature during exercise from rest; HR, heart rate; %HR_max_, percentage of age-estimated maximum heart rate; SR_BSA_, estimated sweat rate relative to body surface area; %BM, percentage of body mass. ^∗^Significantly different from Days 1–4 (p < 0.05).*

### Hot Time Trials

**FIGURE 3:**
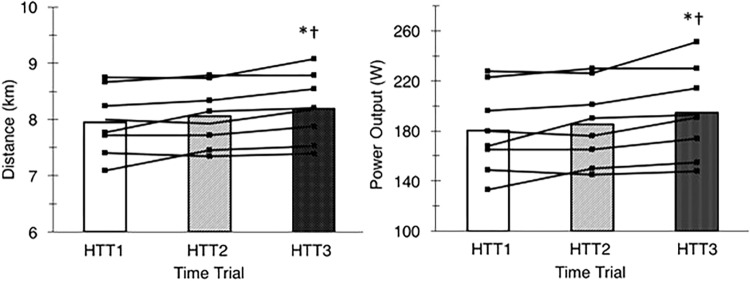
Distance cycled **(left)** and mean power output **(right)** during time trials in hot conditions (35°C, 30% RH) performed pre-acclimation (HTT1), following 4-days heat acclimation (HTT2), and following 9-days heat acclimation (HTT3). Bars represent mean value and black lines represent individual participant data. ^∗^Significant increase from HTT1 (*p* < 0.05); ^†^Significant increase from HTT2 (*p* < 0.05).

There was a large ($\eta_{p}^{2}$ = 0.55) and significant (*p* < 0.01) main effect of acclimation on distance cycled during time trials in hot conditions (Figure 3). *Post hoc* analysis indicated that distance increased by 3.2% (+240 m, [+70, +420]; *p* = 0.01) from HTT1 to HTT3, and by 1.8% (+140 m, [+10, +270]; *p* = 0.04) from HTT2 to HTT3 (Table 3). There was no difference in distance cycled from HTT1 to HTT2 (+100 m, [−100, +300]; *p* = 0.47).

**Table 3:** Performance measures during time trials in the heat (35°C, 30% RH).

	HTT1	HTT2	HTT3
Distance (km)	7.96 ± 0.58	8.06 ± 0.55	8.20 ± 0.59^∗†^
Mean power (W)	180 ± 34	185 ± 32	195 ± 36^∗†^
Mean speed (km ⋅ h^−1^)	31.9 ± 2.3	32.3 ± 2.2	32.8 ± 2.4^∗†^

*Data are presented as mean ± SD. HTT1, time trial pre-acclimation; HTT2, time trial following 4-days heat acclimation; HTT3, time trial following 9-days heat acclimation. ^∗^Significant increase from HTT1; ^†^Significant increase from HTT2, (p < 0.05).*

**FIGURE 4:**
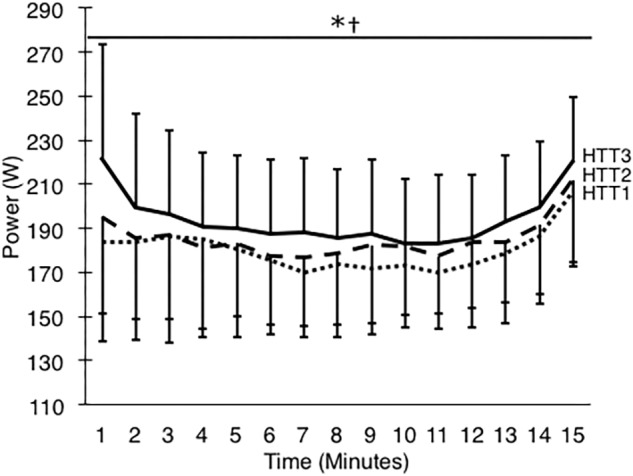
Power output (W) during 15-min time trials in hot conditions (35°C, 30% RH) performed pre-acclimation (HTT1; *dotted line*), following 4-days heat acclimation (HTT2; *dashed line*), and following 9-days heat acclimation (HTT3; *solid line*). Each data point is an average of the preceding minute, and presented as mean ± SD. Significant effect of condition: ^∗^HTT3 > HTT1 (*p* < 0.05), ^†^HTT3 > HTT2 (*p* < 0.05).

These results were matched by the comparison of minute averages of power output. A two-way ANOVA yielded a large ($\eta_{p}^{2}$ = 0.59) and significant main effect of acclimation (*p* < 0.01), but no significant time–condition interaction (*p* = 0.20; Figure 4). *Post hoc* analysis of condition indicated that mean power output across 15-min increased by 8.1% (+14 W, [+4, +24]; *p* = 0.01) from HTT1 to HTT3, and by 4.8% (+9 W, [0, +18]; *p* = 0.05) from HTT2 to HTT3 (Table 3). There was no difference in mean power output from HTT1 to HTT2 (+5 W, [−6, +16]; *p* = 0.55). Additionally, AUC calculated from minute averages of power output yielded a large ($\eta_{p}^{2}$ = 0.58) and significant (*p* < 0.01) main effect of acclimation. *Post hoc* comparisons revealed that power output AUC during HTT3 was greater than in HTT1 (+7.6%, [+2.1, +12.8]; *p* = 0.01) and showed a trend toward increases from HTT2 (+4.4%, [−0.4, +7.7]; *p* = 0.07). Power output AUC was not different between HTT1 and HTT2 (+3.2%, [−3.0, +7.0]; *p* = 0.53).

There was a large ($\eta_{p}^{2}$ = 0.57) and significant (*p* < 0.01) main effect of acclimation on mean cycling speed during time trials in hot conditions. *Post hoc* analysis indicated that mean cycling speed increased by 3.0% (+0.9 km ^⋅^ h^−1^, [+0.2, +1.7]; *p* = 0.01) from HTT1 to HTT3, and by 1.6% (+0.5 km ^⋅^ h^−1^, [0, +1.1]; *p* = 0.05) from HTT2 to HTT3 (Table 3). There was no difference in distance cycled from HTT1 to HTT2 (+0.4 km ^⋅^ h^−1^, [−0.4, +1.2]; *p* = 0.44).

**FIGURE 5:**
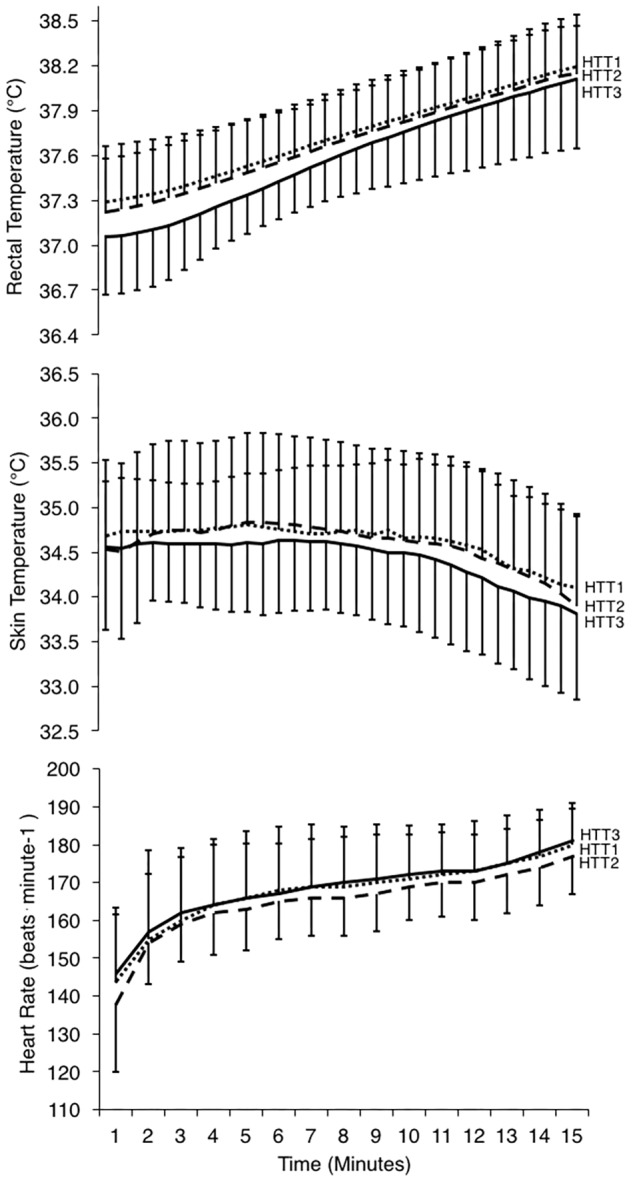
Physiological responses during 15-min time trials in hot conditions (35°C, 30% RH) performed pre-acclimation (HTT1; *dotted line*), following 4-days heat acclimation (HTT2; *dashed line*), and following 9-days heat acclimation (HTT3; *solid line*). Rectal temperatures **(top)** and skin temperatures **(middle)** are displayed as values at 30-s intervals, and heart rate (**bottom**; recorded continuously) is represented as an average of the preceding minute. Data points are presented as mean ± SD.

Mean (*p* = 0.63), peak (*p* = 0.97), and change (*p* = 0.46) in *T*_rec_ during the HTTs were not affected by HA (Table 4). A two-way ANOVA showed no significant main effect of condition (*p* = 0.36) or condition-time interaction (*p* = 0.65) for *T*_rec_ measured each minute of HTTs (Figure 5). There was an average reduction in *T*_rec_ at rest, although this was not significant (*p* = 0.07; Table 4). AUC for *T*_rec_ during HTTs (calculated from minute averages) was not significantly different between HTTs (*p* = 0.39). Mean (*p* = 0.26) and peak (*p* = 0.13) skin temperatures (*T*_sk_) during HTTs were not affected by HA (Table 4 and Figure 5). Mean HR (*p* = 0.48; Figure 5) and mean (*p* = 0.45) and peak (*p* = 0.38) percentage of age-estimated HR maximum (%HR_max_) during HTTs was not different between HTTs (Table 4).

**Table 4:** Physiological and psychophysical measures recorded during time trials in hot conditions.

	HTT1	HTT2	HTT3
**Thermoregulatory**			
Resting *T*_rec_ (°C)	37.2 ± 0.4	37.2 ± 0.3	37.0 ± 0.4
Mean *T*_rec_ (°C)	37.7 ± 0.2	37.7 ± 0.3	37.6 ± 0.3
Peak *T*_rec_ (°C)	38.1 ± 0.3	38.1 ± 0.3	38.1 ± 0.4
Δ*T*_rec_ (°C)	0.9 ± 0.5	0.9 ± 0.3	1.0 ± 0.6
Mean *T*_sk_ (°C)	34.6 ± 0.6	34.1 ± 0.7	34.5 ± 0.9
Peak *T*_sk_ (°C)	35.0 ± 0.5	34.5 ± 0.5	35.0 ± 0.5
**Cardiovascular**			
Mean HR (beats ⋅ minute^−1^)	168 ± 14	165 ± 9	168 ± 12
Mean %HR_max_ (%)	87 ± 7	86 ± 4	87 ± 6
Peak %HR_max_ (%)	94 ± 5	93 ± 5	95 ± 4
**Sudomotor response**			
Active sweat glands per cm^2^	58 ± 23	63 ± 24	75 ± 25^†^
**Blood lactate**			
Pre-test (mmol ⋅ L^−1^)	1.2 ± 0.7	1.0 ± 0.4	1.0 ± 0.5
Post-test (mmol ⋅ L^−1^)	11.1 ± 4.1	11.3 ± 1.7	12.8 ± 2.7
**Thermal comfort (10-point scale)**			
Pre-test	3 ± 2	3 ± 1	2 ± 1
Post-test	5 ± 1	5 ± 2	5 ± 2
**Thermal sensation (13-point scale)**			
Pre-test	9 ± 1	9 ± 1	8 ± 0^∗^
Post-test	10 ± 1	10 ± 1	10 ± 1

*Data are presented as mean ± SD. HTT1, pre-acclimation time trial; HTT2, time trial following 4-days heat acclimation; HTT3, time trial following 9-days heat acclimation. T_rec_, rectal temperature; ΔT_rec_, change in rectal temperature during time trial; T_sk_, weighted mean skin temperature; HR, heart rate; %HR_max_, percentage of age-estimated maximum heart rate. ^∗^Significant difference from HTT1, ^†^significant difference from HTT2, (p < 0.05).*

**FIGURE 6:**
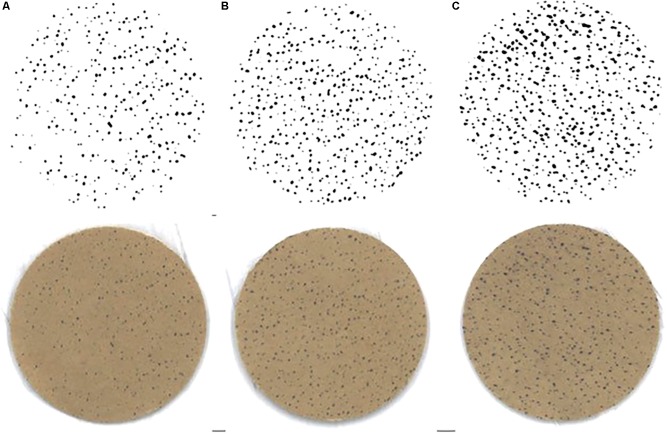
Example of sweat gland activity measured immediately following time trials **(A)** pre-acclimation (HTT1), **(B)** following 4-days heat acclimation (HTT2), and **(C)** following 9-days heat acclimation (HTT3). Bottom row images are scanned copies of iodine-cotton paper applied to participant’s forearm. Top row of images are the same images following computer processing (ImageJ; Gagnon et al., 2012).

There was a significant change in number of active sweat glands immediately following HTTs (main effect: *p* = 0.01, $\eta_{p}^{2}$ = 0.64). *Post hoc* analysis indicated that number of active sweat glands increased by 33% (+17 active sweat glands per cm^2^, [+3, +30]; *p* = 0.02) from HTT1 to HTT3, and by 22% (+12 active sweat glands per cm^2^, [+6, +17]; *p* < 0.01) from HTT2 to HTT3 (Table 4). There was no difference in number of active sweat glands from HTT1 to HTT2 (+5 active sweat glands per cm^2^, [−7, +17]; *p* = 0.62). An example of sweat gland activity recorded following HTTs is depicted in Figure 6.

There was no significant difference in SR_BSA_ during HTTs and including the 75 min of HA that followed (main effect: *p* = 0.08). There were no differences in blood lactate at rest (immediately preceding HTTs; *p* = 0.34) or immediately following HTTs (*p* = 0.41; Table 4). Ratings of thermal sensation taken in the environmental chamber immediately before exercise were significantly different between the HTTs (main effect: *p* = 0.02; Table 4), and on average, corresponded to “Warm” (HTT1), “Warm” (HTT2) and “Slightly Warm” (HTT3). *Post hoc* pairwise comparisons indicated that differences were between HTT1 and HTT3 (*p* = 0.03). There were no significant differences between HTT1 and HTT2 (*p* = 0.10) or between HTT2 and HTT3 (*p* = 0.08). Ratings of thermal comfort taken in the environmental chamber immediately before exercise were not significantly different between HTTs (average ratings corresponded to ratings between “Comfortable” and “Slightly Uncomfortable”; *p* = 0.39; Table 4). Ratings of thermal sensation and thermal comfort taken at the end of HTTs were not significantly different between HTTs (“Hot” [*p* = 0.25] and “Uncomfortable” [*p* = 0.53], respectively; Table 4).

## Discussion

This study was designed to determine whether STHA (4-days) is sufficient to improve self-paced endurance performance in hot conditions in females, as has been observed in males, or whether a longer heat acclimation stimulus (i.e., 9-days) is required. In this study’s female cohort, STHA did not significantly improve time-trial performance in the heat; however, 9-days HA did. These results were consistent with the study hypothesis, which predicted that STHA would be insufficient to improve self-paced performance in females, and that a longer heat acclimation stimulus would be required to induce the physiological adaptations needed for performance improvements in the heat.

### Self-Paced Endurance Performance

Following STHA, female participants showed no significant performance improvements in distance cycled, mean power output, or speed during HTT2, as compared to HTT1. This is in direct contrast to a number of studies in male cohorts, where males have shown meaningful physiological adaptions *and* improved endurance performance in the heat following STHA (Garrett et al., 2009, 2012; Chen et al., 2013; Racinais et al., 2015; Guy et al., 2016; James et al., 2016; Willmott et al., 2016; Wingfield et al., 2016). Thus, it appears that STHA using 90 min of daily exercise heat stress is insufficient to improve endurance performance in females, reflecting the lack of physiological adaptation to heat acclimation previously demonstrated in females following STHA (Mee et al., 2015). The current study’s performance results following STHA differ from those observed by Sunderland et al. (2008), who reported a 33% improvement in distance run during a repeated shuttle run performance test (Loughborough Intermittent Shuttle Test) following STHA (4-days) in a female cohort. Of note, time to exhaustion is the main outcome measure of the Loughborough Intermittent Shuttle Test. This outcome is influenced by technique (i.e., ability to change direction and accelerate; Mendez-Villanueva and Buchheit, 2013), making it less reliable and subject to greater variation than the self-paced performance trial used in the current study (Hickey et al., 1992; Gosens et al., 2015; Borg et al., 2018). Furthermore, the behavioral regulation of performance possible in a self-paced time trial is not available in a time to exhaustion protocol (Schlader et al., 2011). Indeed, the lower pre-exercise thermal sensation reported by participants after 9-days HA may be an indication of perceptual changes contributing to behavioral regulation (i.e., pacing). Thus, the self-paced performance test used in the current study is a more reliable and holistic assessment of performance than a time to exhaustion test. Despite efforts in the current study to create an “intense” heat stimulus by combining isothermic heat acclimation, HIIT, and permissive dehydration, it still appears that females require either a longer daily heat exposure (Mee et al., 2018), or a greater number of heat exposures (as observed in the current study and by Mee et al., 2015) to improve exercise performance in the heat.

This is the first study to quantify improvements in self-paced time trial performance following a longer (i.e., 9-days) heat acclimation stimulus in a female cohort. The ∼8% mean improvement in mean power output in HTT3 as compared to HTT1 is comparable to performance improvements observed in male, or mostly male cohorts following similar heat acclimation protocols. Keiser et al. (2015) showed that male participants experienced a ∼10% improvement in power output during a 30-min self-paced time trial following 10-days heat acclimation (daily bouts: 90-min cycling at 50% VO_2max_ in 38°C, 30%RH). Lorenzo et al. (2010) also found that participants (10 males and 2 females) had an 8% mean improvement in power output during their 1-h self-paced time trial following 10-days heat acclimation (daily bouts: 90-min cycling at 50% VO_2max_ in 40°C, 30%RH). In the current study, improvements in mean power output coincided with improvements in mean cycling speed and distance covered from HTT1 to HTT3. These data demonstrate that 9-, but not 4-days heat acclimation, improves endurance performance outcomes in females.

### Physiological Measures

Participants exhibited reduced markers of physiological strain (i.e., *T*_rec_, *T*_sk_ and HR) during days 5–9 of HA, as compared to days 1–4. These physiological changes occurred in spite of an increased mean power output during days 5–9 of HA. Although these data indicate a reduction in the desired stimulus across the heat acclimation protocol, it also indicates that the greatest heat stimulus was administered during STHA. Furthermore, the improved performance in HTT3 as compared to the previous HTTs indicates that this reduced stimulus during days 5–9 was still effective in producing HA-related performance improvements. Also, this HA protocol produced a sufficient dehydration stimulus, as the ∼3% body mass loss achieved across HA days 1–4 and 5–9 in addition to permissive dehydration presumably exceeded the osmotic threshold required for compensatory fluid regulatory responses (i.e., 2% body mass loss; Cheuvront and Kenefick, 2014). However, as we did not measure changes in plasma volume, it is unknown whether participants experienced the fluid regulatory responses typically associated with heat acclimation.

There was a trend for a lower *T*_rec_ at rest before HTT3, which appeared to influence *T*_rec_ during the initial minutes of HTT3 (albeit not significantly). Menstrual cycle phase and associated changes in female sex hormones influence resting *T*_rec_ (Inoue et al., 2005) and the overall thermoregulatory set point range (Charkoudian and Stachenfeld, 2016). This may have contributed to the non-significant change in resting *T*_rec_ observed in the current study. By the end of each HTT, *T*_rec_ reached similar values (∼38.1°C). This is perhaps unsurprising as a previous study has shown that heat acclimation does not change the maximal *T*_rec_ reached (40.1–40.2°C) during a 43.4-km time trial in the heat, despite a lower *T*_rec_ for the first 80% of the post-acclimation time trial (Racinais et al., 2015).

In the current study, there was an observed increase of active sweat glands at the end of HTT3 (Table 4). This contrasts findings in male cohorts, where sweat gland activation did not increase following 8–10-days heat acclimation (Inoue et al., 1999; Lee et al., 2010; Poirier et al., 2016). In the current study, the number of active sweat glands (75 ± 25 per cm^2^) at the end of HTT3 were lower than values previously reported in acclimated males (∼96–108 per cm^2^; Inoue et al., 1999; Lee et al., 2010; Poirier et al., 2016) and unacclimated females (∼93 per cm^2^; Knip, 1969). Therefore, changes observed following a 15-min HTT may not indicate improved maximal sweat gland activation *per se*, but rather earlier activation of the sweat glands. Although there is large intra-subject coefficient variation associated with this measure, the 33 and 22% mean improvements following HTT3 in comparison to HTT1 and HTT2, respectively, surpass the ∼11% coefficient of variation reported by Gagnon et al. (2012).

### Perspectives

These results contribute to the limited research that informs the expected performance outcomes of heat acclimation for female athletes. The results of this study indicate that while heat acclimation can be an effective training component in preparation for competition in the heat, female athletes may require up to 9 days of 90-min heat acclimation sessions before experiencing performance improvements. However, there will be individual variation in how athletes (male or female) respond to heat acclimation (Racinais et al., 2012). In the current study, three participants’ performance deteriorated in HTT2 as compared to HTT1, whereas four participants showed improvements and one participant showed no change. Thus, some female athletes may achieve meaningful performance benefits after 4-days heat acclimation, while others could require longer than 9-days. A heat acclimation protocol lasting longer than 9-days has yet to be initiated in a female cohort, which would be hypothesized to further stabilize adaptions and improve performance (Racinais et al., 2015). It is also unclear how different phases of the menstrual cycle/contraception may affect heat adaption during acclimation. Future research is also needed to clarify the impact of mixed-intensity heat acclimation on longer performance tests in both male and female athletes.

### Considerations

Despite the absence of a control group, it is unlikely that performance improvements in HTT3 were due to learning or training effects. After preliminary testing and familiarizations, HTT1 was the fourth time that participants would have completed the 15-min time trial, minimizing learning effects. Furthermore, performance improvements in the current study are similar to previous studies (Lorenzo et al., 2010; Keiser et al., 2015), where control groups showed no improvements.

It is possible that the high-intensity heat acclimation protocol used in the current study may have caused a general fatigue that impaired performance during HTT2 and HTT3 (Schmit et al., 2018; Reeve et al., 2019). However, this is a negative bias as fatigue-related performance impacts would presumably have been greatest at HTT3. A further consideration is that heat acclimation adaptions are specific to the type/intensity of exercise employed (Wingfield et al., 2016). Therefore, the 15-min of HIIT undertaken at the beginning of each HA session may have facilitated specific adaptations. Whether this type of mixed-intensity heat acclimation (15-min HIIT + 75-min isothermic HA) would be equally or more effective than steady-state isothermic heat acclimation protocols typically reported in the literature remains unknown.

This study did not control for menstrual cycle. Recent data has shown that performance under heat stress is not affected by menstrual cycle or oral contraceptive pill (OCP) use in trained female athletes (Lei et al., 2017, 2018), nor does menstrual cycle affect whole-body heat loss (Notley et al., 2018). Eumenorrheic participants and OCP users did not cross over phases between HTT1 and HTT2. Participants were counterbalanced in their phases in HTT3, with both eumenorrheic participants being in opposite phases and both OCP users being in opposite phases (i.e., pill-taking, or non-pill-taking). None of the other four participants [contraceptive implant or copper intrauterine device (IUD)] were menstruating during the protocol, mitigating concerns of premenstrual symptoms that could affect performance (Giacomoni et al., 2000). Despite this, variable hormonal states may have affected the degree of relative heat stimulus administered when targeting an absolute core temperature of 38.5°C during heat acclimation sessions.

Finally, it should be noted that measures of sweat gland activity were taken from sites on the forearm and are not a precise indication of whole-body sweat gland adaptations given the regional heterogeneity of sweat gland activity. While an increased sweat gland activity may imply a better use of body surface area to dissipate heat, sweat gland activation is not directly proportional to local sweat output of the area (Poirier et al., 2016). In future, measures of local sweat output should be combined with measures of sweat gland activation to fully understand sex differences in peripheral sudomotor adaptations.

## Conclusion

This study was the first to document performance outcomes during self-paced time trials in a female cohort following STHA (4-days) and 9-days high-intensity, isothermic HA. In the current study, females did not show an improvement in self-paced endurance performance following STHA. This differs from the well-documented performance improvements previously observed in male cohorts following STHA. However, following 9-days HA, females achieved meaningful improvements in self-paced endurance performance. These improvements included an ∼8% increase in mean power output, a ∼3% increase in distance cycled, and a ∼3% increase in speed when performing a 15-min self-paced time trial in hot conditions (HTT). These data offer a reference for the changes which female athletes can expect when undergoing heat acclimation with the aim of improving self-paced endurance exercise performance in hot conditions, and provides further evidence that STHA may be insufficient for female athletes.

## Ethics Statement

This study was carried out in accordance with the recommendations of the University of Birmingham Ethics Committee with written informed consent from all subjects.

## Author Contributions

NK, SL, and RL contributed to the conception and design of the study, acquisition, analysis, and interpretation of the data, drafted, revised, and approved the final version of the manuscript.

## Conflict of Interest Statement

The authors declare that the research was conducted in the absence of any commercial or financial relationships that could be construed as a potential conflict of interest.

## Abbreviations

%HR_max_: percentage of age-estimated maximum heart rate
$\eta_{p}^{2}$: partial eta-squared
AUC: area under the curve
BSA: body surface area
HA: heat acclimation sessions
HIIT: high-intensity interval training
HR: heart rate
HTT: time trial in hot conditions
IUD: intrauterine device
NBM: nude body mass
OCP: oral contraceptive pill
SR_BSA_: estimated sweat rate relative to body surface area
STHA: short-term heat acclimation
Sweat Loss_%BM_: estimated sweat loss as a percentage of body mass
*T*_rec_: rectal temperature
*T*_sk_: weighted mean skin temperature
VO_2peak_: maximal aerobic capacity
W: watts.
